# Moving towards the implementation of pharmacogenetic testing in Quebec

**DOI:** 10.3389/fgene.2023.1295963

**Published:** 2024-01-03

**Authors:** Ling Jing Li, Samuel Legeay, Ann-Lorie Gagnon, Marie-Pier Frigon, Laurence Tessier, Karine Tremblay

**Affiliations:** ^1^ Centre Intégré Universitaire de Santé et de Services Sociaux Du Saguenay-Lac-Saint-Jean (Chicoutimi University Hospital), Research Center, Saguenay, QC, Canada; ^2^ Medicine Department, Faculty of Medicine and Health Sciences, Université de Sherbrooke, Saguenay, QC, Canada; ^3^ University Angers, [CHU Angers], Inserm, CNRS, MINT, Angers, France; ^4^ Pediatrics Department, Faculty of Medicine and Health Sciences, Université de Sherbrooke, Sherbrooke, QC, Canada; ^5^ Pharmacology-Physiology Department, Faculty of Medicine and Health Sciences, Université de Sherbrooke, Saguenay, QC, Canada; ^6^ Centre de Recherche Du Centre Hospitalier Universitaire de Sherbrooke (CR-CHUS), Sherbrooke, QC, Canada

**Keywords:** pharmacogenetic testing, pharmacogenomics, precision medicine, clinical implementation, challenges pharmacogenetics testing implementation in Quebec

## Abstract

Clinical implementation of pharmacogenetics (PGx) into routine care will elevate the current paradigm of treatment decisions. However, while PGx tests are increasingly becoming reliable and affordable, several barriers have limited their widespread usage in Canada. Globally, over ninety successful PGx implementors can serve as models. The purpose of this paper is to outline the PGx implementation barriers documented in Quebec (Canada) to suggest efficient solutions based on existing PGx clinics and propose an adapted clinical implementation model. We conclude that the province of Quebec is ready to implement PGx.

## Introduction

The field of pharmacogenetics (PGx) holds the promise to personalize clinical practice using genetic variants to optimize treatment, prevent adverse drug reactions (ADRs), and reduce avoidable costs for the healthcare system and patients. Indeed, studies show that approximately 91%–99% of the population carry at least one actionable pharmacogenetic variant, which is defined as being clinically significant in therapeutic efficacy or risk of ADRs (Van Driest et al., 2014; Van Der Wouden et al., 2017; Mroz et al., 2021; Tafazoli et al., 2021; Morris et al., 2022; Huang et al., 2023; Kabbani et al., 2023; Oni-Orisan et al., 2023). To date, a large number of genetic variants have demonstrated a clinical utility and are incorporated for genetic testing into at least 430 drug labels (including 130 drugs used in oncology) by the United States Food and Drug Administration (FDA) and 182 by Health Canada (PharmGKB, 2023). Clear guidelines on these relevant drug-gene interactions and their therapeutic recommendations have been published by the Clinical Pharmacogenetics Implementation Consortium (CPIC) and the Dutch Pharmacogenetics Working Group (DPWG) (Relling and Klein, 2011; Swen et al., 2011; CPIC, 2023; PharmGKB, 2023). Within the past decades, over ninety PGx testing clinics aiming to implement PGx tests into routine clinical care emerged worldwide (CPIC, 2023; Genome.gov, 2023b). In Europe, the Ubiquitous Pharmacogenomics Consortium (U-PGx) has successfully implemented a multidrug, multigene, multicenter and multi-ethnic approach for PGx (Van Der Wouden et al., 2017). In Asia, the South East Asian Pharmacogenomics Research Network (SEAPharm) has established a large program in five Asian countries to make PGx testing available for these populations (Chumnumwat et al., 2019). In the United States of America (USA), around 70 institutions implement CPIC guidelines clinically, with some programs over decade-long, demonstrating promising results (Krebs and Milani, 2019; CPIC, 2023). Even if we consider the population ratio between the USA and Canada, Canada’s PGx implementation is progressing at a slower pace with some sparse implementers such as the Genome British Columbia Center in the past (Genome BC, 2023). As of September 2023, in the list of implementers of the CPIC guidelines, only four institutions are Canadian (CPIC, 2023): the Hospital for Sick Children at Toronto [multi-gene panel implementation pilot study (SickKids, 2023)], the University of Alberta [implementation pilot study for psychiatry drugs (University od Alberta, 2023)], the University of Calgary [Pharmacogenetic-Supported Prescribing in Kids (PGx-SParK), a pilot study in psychiatry (University of Calgary, 2023)], and the Sunnybrook Health Sciences Centre at Toronto [PGx testing (Sunnybrook Hospital, 2023)].

To our knowledge, while there have been a couple of research teams who have worked on the perceptions of healthcare providers, pharmacists and patients regarding pharmacogenomics implementation in Quebec (Canada) (De Denus et al., 2013; Frigon et al., 2019; Meloche et al., 2020; Petit et al., 2020; Subasri et al., 2021), few have proposed an implementation model adapted toward the specific needs of the Quebec healthcare system. This entails a prolonged implementation program to facilitate and integrate genetic testing to guide patient prescriptions in clinical settings. Naturally, this proposed model should first be evaluated by research projects and then implemented in the healthcare system on a broader scale. Herein, we will discuss solutions to the specific challenges of clinical PGx implementation in Quebec by referencing successful existing PGx clinical implementation models. The solutions will serve to propose a model of clinical implementation adapted to the province’s community, resources, and perspectives.

## Contextualization of the Quebec healthcare system

The Quebec healthcare system, like the rest of Canada, differs greatly from its American counterpart. It is a public system following a single-payer, publicly funded model meaning that the government is the primary payer through tax revenue. This entails that all residents have access to universal coverage, extending from essential medical services to prescription drugs through programs like the “*Régie de l’assurance maladie du Québec*” (RAMQ). At a provincial level, the “*Ministère de la Santé et des Services Sociaux*” (MSSS) oversees healthcare policies, decision-making and coordination. Directly under, on a regional level, institutions such as “*Centre Intégré Universitaire de Santé et de Services Sociaux*” (CIUSSS) amongst many others are responsible for regulated and consistent healthcare delivery. This centralized system ensures a standardized quality of care throughout the province and a certain equity in healthcare accessibility (Quebec, 2023). This also facilitates the existence of shared electronic medical records (EMRs) across institutions such as the “*Dossier Santé Québec*” (DSQ). The DSQ is a centralized communication platform which stores patients’ health information (e.g., prescribed medications, laboratory test results, and medical imaging results) and facilitates its secure and timely sharing between authorized organizations and stakeholders to improve efficiency, coordination, and quality of care (Dossier santé Québec DSQ, 2023).

## Previously raised challenges and efficient solutions

The costs, the lack of easily accessible guidelines, the ethical and insurance issues, and the lack of educational resources for clinicians were the main barriers associated with PGx implementation perceived by Quebec primary care physicians (PCPs), pharmacists and patients (Frigon et al., 2019). The next subsections present solutions to overcome these barriers raised by previous studies (De Denus et al., 2013; Frigon et al., 2019; Meloche et al., 2020; Petit et al., 2020; Subasri et al., 2021).

### Cost associated with PGx testing

The cost of PGx testing is seen as the greatest challenge to its implementation in Quebec (Frigon et al., 2019). However, meta-analyses have shown that a majority of PGx-guided treatments are cost-effective or even cost-saving (Verbelen et al., 2017; Karamperis et al., 2021; Morris et al., 2022; Kabbani et al., 2023). For example, two studies mentioned cost-savings from sources such as reduction in medication or reduction of hospitalization from drug toxicity between 1036 USD to 3962 USD from PGx-guided therapy per patient per year even with test costs considered (Winner et al., 2015; Kabbani et al., 2023). The cost of a single gene test ranges from 100 to 500 USD while a panel test by microarray could reach double the price (Kabbani et al., 2023). In Canada, the cost of PGx testing can range from 199 to 2310 CAD (MBA VL MSc Medicinal Chemistry, 2016; Maruf et al., 2020). Therefore, long-term, a pre-emptive genotyping approach coupled with panel-based testing yields more cost-effectiveness throughout a patient’s medical care by reducing the number of single-gene tests (ODonnell et al., 2012; Bielinski et al., 2014; Van Driest et al., 2014; Klein et al., 2017; Blagec et al., 2022; Hayashi et al., 2022; Morris et al., 2022; Huang et al., 2023; McDermott et al., 2023; Swen et al., 2023) by 60% (Van Driest et al., 2014; Krebs and Milani, 2019). The pre-emptive approach however entails that all patients undergo the panel test. This can be considered as a limitation of the implementation due to its high cost and the need for a positive attitude and willingness on the patients’ part towards PGx tests.

Furthermore, with rapid technological development, newer technologies and approaches emerge such as Next-Generation Sequencing (NGS) and the numerous approaches in utilizing this platform, e.g., whole-exome sequencing (WES), whole-genome sequencing (WGS) or targeted sequencing (Tafazoli et al., 2021). A study in China with 22,918 participants across 20 provinces using a 52-gene targeted NGS PGx panel successfully reduced the sequencing cost of their panel-based test to a few US dollars per sample (Huang et al., 2023). Also, WES and WGS, while costly with prices for WGS ranging in the thousands and WES, around a quarter of that price (Schwarze et al., 2018), are great choices in the study and identification of novel biomarkers while simultaneously repurposing the information to extract a PGx profile for the clinician (Tafazoli et al., 2021). NGS technology is readily available in Quebec at organizations such as Genome Quebec (Sequencing, 2023). While this technology is progressively becoming more frequently utilized and accessible, there still exist many limitations. Several PGx variants are outside of WES’s usual captured regions and genes with many variants are currently a challenge for NGS technology, for example, (Tafazoli et al., 2021). Beyond the cost of testing, clinical NGS testing needs to factor in the cost and management of bioinformatic infrastructures and services, software and trained personnel to name a few (Tafazoli et al., 2021). Despite this, we believe it can serve as a promising direction for the future.

Lastly, the savings achieved by PGx-guided treatment can help cover the cost of the machines, testing or biobanking, for example. In a microsimulation model of British Colombia (BC) to evaluate the effectiveness and cost-effectiveness of PGx-guided treatment for major depressive disorder over 20 years, the expenditure, even accounting for PGx testing of 738 CAD (average cost of available PGx tests in Canada), is offset by the decrease in the cost of refractory care (37% decrease) with a cost-saving of 4926 CAD per patient as well as gains of 0.064 life-years (Ghanbarian et al., 2023).

### Role of healthcare providers in PGx testing

Quebec PCPs and pharmacists are concerned about their role in the implementation of PGx testing (De Denus et al., 2013; Frigon et al., 2019). Due to their expertise in the optimal use of medications, most healthcare professionals (HCPs) from primary care and patients surveyed agreed pharmacists should play a central role (De Denus et al., 2013; Frigon et al., 2019; Meloche et al., 2020; Petit et al., 2020). However, as PGx is shifting towards a pre-emptive approach, with recommendations in patients’ EMRs, PCPs are at the forefront of PGx implementation (ODonnell et al., 2012; Pulley et al., 2012; Gottesman et al., 2013; Johnson et al., 2013; Hoffman et al., 2014; Shuldiner et al., 2014; Caraballo et al., 2017; Rosenman et al., 2017; Aquilante et al., 2020; Duarte et al., 2021; Hayashi et al., 2022; Kabbani et al., 2023; McDermott et al., 2023). Recently, more multidisciplinary approaches have been proposed (Calinski et al., 2021; Loudon et al., 2021; Genome.gov, 2023a). For example, the Genome Education Resource Center (GenomeEd) defines roles in PGx implementation for pharmacists as the drug-gene experts who synthesize relevant PGx information and monitor PGx-guided plans, while physicians are responsible for recognizing potential drug-gene interactions in patient records and for consulting other healthcare professionals such as pharmacists (Korf et al., 2014; Gammal et al., 2022; Genome.gov, 2023a). The preemptive approach allows HPCs to directly adapt drugs’ prescription at the time of prescription using the readily available results of the patient’s panel genotyping and, consequently, protect patients from ADRs. Furthermore, a survey with genetic counsellors in North America proposes pharmacogenomic counselling be handled by both genetic counsellors and pharmacists (Loudon et al., 2021). We thus strongly believe that the implementation of PGx testing should be multidisciplinary. PGx data integration into EMR should be easily available in all institutions.

### Lack of clinical guidelines

HCPs from primary care commonly report a lack of clear and accessible clinical guidelines hindering the implementation of PGx in their clinical practice (Frigon et al., 2019). However, the CPIC and the DPWG provide freely accessible, evidence-based, peer-reviewed, and updated PGx clinical guidelines to support healthcare professionals in implementing PGx into their practice (Relling and Klein, 2011; Swen et al., 2011). As of late 2022, the CPIC has published 26 guidelines while the DPWG has published 47 guidelines (McDermott et al., 2023; Oni-Orisan et al., 2023). This situation suggests communication issues between scientific committees and HCPs. Therefore, there is a need to bridge the gap between the guidelines mentioned above and clinical practice (Maruf and Bousman, 2022). Noteworthy expert associations or networks, e.g., the International Society of Psychiatric Genetics (ISPG), can issue clinically adapted recommendations such as guidance on the use of PGx testing in their domain of expertise (Maruf and Bousman, 2022). At the provincial level, Quebec government entities such as the « *Institut national d’excellence en santé et services sociaux* » (INESSS) can also develop clinical tools (e.g., DPYD and chemotherapy toxicity) (INESSS, 2023). These clinical tools should however be adapted into clinical decision support (CDS) alerts to facilitate the adherence of HCPs to the guidelines.

To enable real-time efficient clinical usage, relevant investments must be made to allow the incorporation of CDS alerts in the EMR and provide clinicians with PGx-guided recommendations at the time of prescription (Frigon et al., 2019). Successful integrations include the PREDICT program at the Vanderbilt University Medical Center and St Jude Children’s Hospital (Van Driest et al., 2014; Maruf and Bousman, 2022). Similarly, U-PGx indicates the feasibility of a standardized multinational, multi-language, and muti-center CDS solution (Van Der Wouden et al., 2017; Blagec et al., 2022), aspects relevant in a bilingual province. Eventually, the cost of systematically installing CDS in centers and clinics could be borne by the savings generated by the reduction in ADR-induced care costs. Coupled with other benefits of CDS such as helping HCPs integrate new guidelines or improving healthcare efficiency and safety (Sutton et al., 2020), public decision-makers are consequently encouraged to support the implementation of CDS and the development of a centralized system facilitated by an already centralized healthcare system.

### Ethical and insurance-related considerations

Accessible and complete genetic data raises concern over the ethical and medicolegal aspects of PGx implementation and most genetic companies or institutions require consent for PGx testing (De Denus et al., 2013; Frigon et al., 2019). For example, incidental findings that can be found with NGS may cause anxiety in patients. Also, sensitive information may fall into the wrong hands, such as insurance companies (De Denus et al., 2013; Frigon et al., 2019). Therefore, education, counselling and robust policies become crucial to empower and protect patients as well as HCPs (Hayashi et al., 2022; Pinzón-Espinosa et al., 2022). The Genetic Non-Discrimination Act (GNA) makes it a criminal offence for a service provider or anyone entering into a contract with a person to require or compel that person to take or disclose the results of, a genetic test (Branch, 2017). This law needs to be made aware as it prevents, as seen in our American counterpart, social discrimination using genetic information by individuals or organizations such as insurance companies (De Denus et al., 2013; Branch, 2017; Hayashi et al., 2022; Oni-Orisan et al., 2023). Continuing with ethical considerations, to guarantee a secure, interoperable and accessible centralized database and digital ecosystem (Albalwy et al., 2022), a multitude of experts and committees are needed. Well-established Quebec organizations such as “*Plateforme de recherche, de valorisation, d’analyse et de liaison en informatique de la santé*” (PREVALIS), a platform developed by the Université de Sherbrooke (Quebec, Canada), provide services to develop such informatics tools as it is comprised of experts in the domains of informatics, artificial intelligence, health, pedagogy and ethics (PREVALIS, 2023). Eventually, the question of alleles that are prevalent in certain ethnic minorities must be solved by expert scientific committees such as CPIC and CPWG. These pertinent alleles should be included in the genotype panel for inclusivity. Awareness-raising campaigns should be conducted to inform all sociodemographic groups about the test and reassure them that there is no risk of harm. Lastly, Quebec’s public healthcare system contributes to enhancing equity in healthcare accessibility.

### Lack of educational resources

PCPs and pharmacists agree that appropriate training remains a primordial step before the implementation of PGx into their practice (De Denus et al., 2013; Frigon et al., 2019; Meloche et al., 2020; Petit et al., 2020). To address this barrier for practitioners, e-learning and web-based education are believed to be the methods of choice (De Denus et al., 2013; Meloche et al., 2020). In Canada, studies like the past Pharmacists: Personalized Medicine Experts (PRIME) PGx prospective cohort study had online modules, training workshops, and simulated patient cases as a formative assessment as part of their continuing professional development program (Crown et al., 2020; McDermott et al., 2023). Otherwise the Pharmacy Association of Nova Scotia (PANS) provides nonaccredited online courses on PGx for community pharmacists (Online Course Registration, 2023).

For future professionals, interprofessional education (IPE) has been recently proposed as an alternative (Calinski et al., 2021; Lee et al., 2023). An example would be the collaboration between the College of Pharmacy at Ferris State University (USA) and the medical program at Western Michigan University (USA) to develop a telehealth session pairing first-year medical students with third-year pharmacist students to solve PGx cases (Lee et al., 2023). IPE experience significantly enhances PGx knowledge and confidence in applying PGx in clinical cases for a majority of medical and pharmacist students as well as increases awareness of each other’s disciplines and improves interprofessional communication and collaboration (Calinski et al., 2021; Lee et al., 2023). Since Quebec has four medical programs alongside two pharmacist programs with some within the same institution (Canadian Pharmatics Association, 2023; Royal College, 2023), IPE sessions should remain feasible. Universities such as McGill (Quebec, Canada) already carried out health-related IPE projects in the past with students from dietetics, genetic counselling, medicine, nursing, and many more (Office of Interprofessional Education, 2023). This method would also aid our view of a multidisciplinary approach to PGx.

### Speed of PGx testing results

Quebec PCPs and pharmacists agreed that PGx test results must be obtained rapidly to be valuable (Frigon et al., 2019). It is unanimous, in literature, that a pre-emptive model of genomic testing has the added benefit of timelessness as genetic data does not change (ODonnell et al., 2012; Johnson et al., 2013; Bielinski et al., 2014; Hoffman et al., 2014; Rasmussen-Torvik et al., 2014; Van Driest et al., 2014; Rosenman et al., 2017; Van Der Wouden et al., 2017; Chumnumwat et al., 2019; Duarte et al., 2021; Albalwy et al., 2022; Hayashi et al., 2022; Morris et al., 2022; Huang et al., 2023; Kabbani et al., 2023; McDermott et al., 2023; Oni-Orisan et al., 2023). Except for the first prescription of a PGx test, there is no waiting period in therapeutic decision-making. Once available, PGx results are added to the patient’s EMRs to be easily consulted by the prescribers. At the time of prescribing a high-risk drug, PCPs receive a CDS alert containing the patient’s genotype and drug response phenotype alongside the appropriate and updated guideline-based recommendations. Thus, in line with the pre-emptive approach, panel genotyping can be realized in children but requires parental consent in this case.

## Potential Quebec model of PGx clinical implementation

**FIGURE 1:**
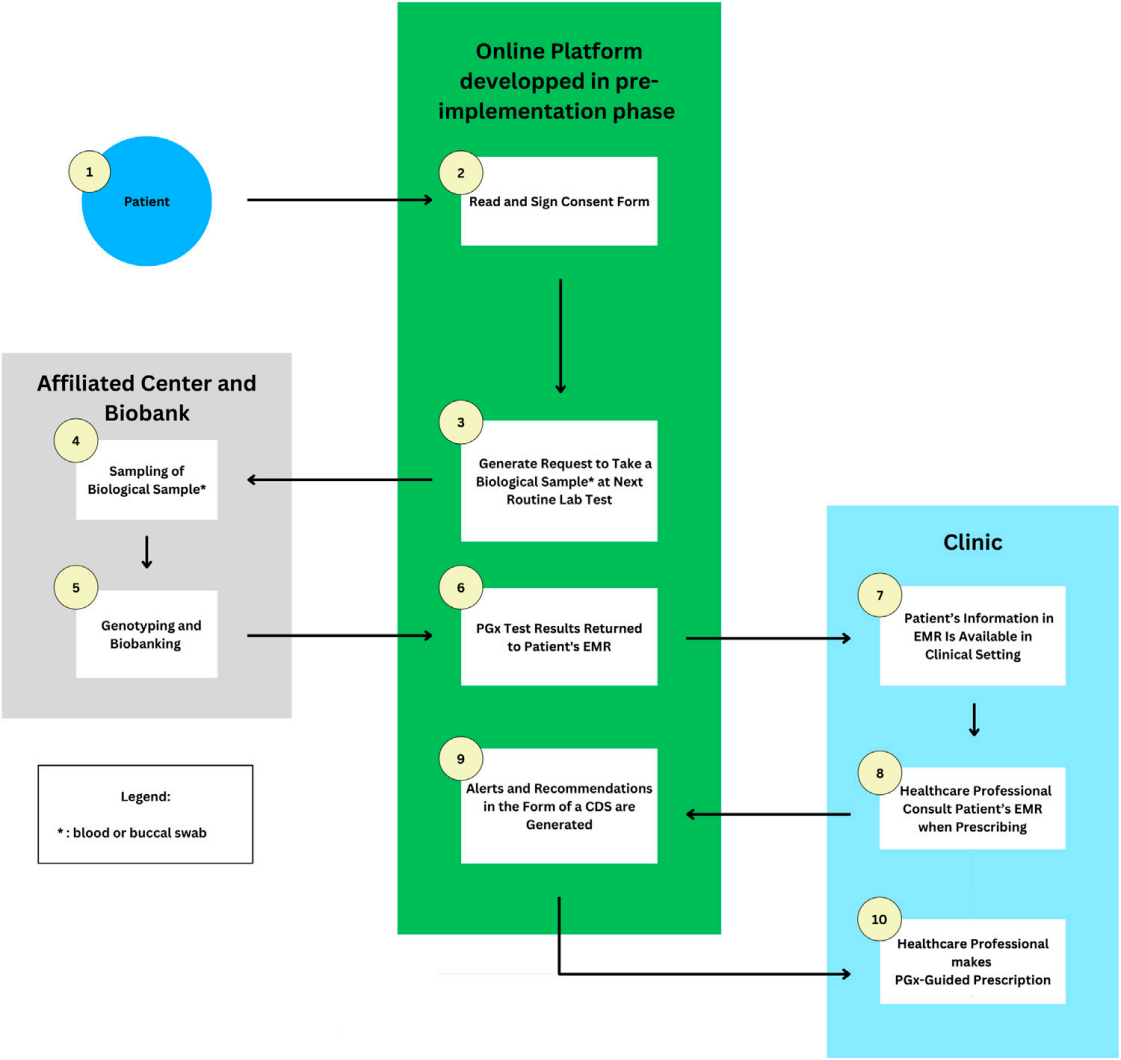
Flowchart of an example of how PGx may be implemented in the Quebec province setting. 1 and 2) Patients give their consent for research and pre-emptive panel based PGx testing on the developed online platform. 3) A request to take a blood sample is emitted when the patient goes for a routine blood test at an affiliated clinic. 4 and 5) The sample is sent to a biobank for genotyping and biobanking. 6) Results are returned to the EMR and online platform. 7–10) Healthcare professionals access relevant information and prescribe medications based on pharmacogenomic-guided recommendations received through CDS alerts. Abbreviations used: PGx, Pharmacogenetic; CDS, Clinical Decision Support; EMR, Electronic Medical Record.

Using the above overview of potential solutions to the major barriers of PGx clinical implementation as a basis, the following sections present the model we believe to be implementable in the Quebec clinical setting in the near future (Figure 1). This model encapsulates the cumulation of the solutions found to address the barriers. Further experimentations on the model are necessary to identify the most suitable implementation model for the Quebec healthcare system. For this model, the PGx implementation should be divided into two phases: the pre-implementation and the implementation phases.

### Pre-implementation phase

The pre-implementation phase aims to define and create the essential foundations for the successful implementation of PGx in clinics such as allowing the formation of key committees and institutions or the creation of required tools to remediate the previously raised barriers. Based on a subsection above, to improve efficacy and cost-effectiveness, Quebec PGx clinics must be preferably pre-emptive and panel-based while utilizing pre-existing resources for its implementation.

The establishment of a multidisciplinary committee of experts in the fields of medicine, pharmacogenetics, ethics, or decision-makers, among other stakeholders, is a first step. Such committee should meet regularly to discuss the implementation of PGx including its maintenance and improvement. Identifying and evaluating gene-drug pairs to implement based on the available evidence-based guidelines and the prevalence of the genetic variation in the population, thus defining the target population in the heterogenous Quebec province for the selected PGx test-panels, will be one of its main goals. Furthermore, this committee will also work towards the acceptance and support of these guidelines by the INESSS to increase the acceptance of PGx in clinicians’ practice. Participation of HCPs, pharmacists, patients, experts in ethics and legislation and decision-makers is mandatory to solve medicolegal aspects of PGx implementation and improve the acceptability of PGx testing.

To address the lack of educational resources, the first step would be to identify the types of training needed by each HCPs for the efficient and multidisciplinary implementation of PGx. For this, semi-structured focus group interviews will have to be conducted. Educational resources could then be accessible from pre-existing online platforms for continuing formation. Universities should also be encouraged to organize online or in-person IPE workshops to improve knowledge of PGx and interprofessional collaboration in future practitioners.

A major effort in the pre-implementation phase will be the development of informatic tools such as a digital ecosystem to facilitate the storing and safe transferring of PGx recommendations and patients’ data between institutions and towards a CDS system to optimize clinician efficiency in applying PGx into their practice. The usage of existing resources such as PREVALIS can speed up the developmental process. Comprised of experts in multiple domains, their expertise in data foraging, data harmonization and artificial intelligence to develop informatic tools in the domain of health would aid in the development of an adapted online platform able to interconnect data from several sources while facilitating its retrieval and integration elsewhere (e.g., EMRs). With experts in ethics already on the board, PREVALIS can also guarantee the medicolegal and ethical aspects of its products (PREVALIS, 2023).

Lastly, to avoid social inequalities and increase usage of PGx testing, Quebec’s public health insurance system should preferably refund the cost of PGx testing. Indeed, a study in Italy shows a doubling of patients pre-emptively tested for *DPYD* the year the test is reimbursed by the government (Bignucolo et al., 2023). However, governmental coverage will require pre-existing proofs of clinical utility, and therefore, funding will be required in the implementation of the first tests to demonstrate clinical utility and increase social acceptance (Klein et al., 2017).

Summarily, at the end of a pre-implementation phase, the foundations for PGx implementation can be established with the formation of effective committees, the identification of the most relevant drug-gene pairs, the conception of appropriate educational resources along with the development of an online platform to integrate, transfer and retrieve PGx information, patient data and CDS. A stable foundation will allow the Quebec PGx program to efficiently adapt and improve with new findings.

### Clinical implementation phase

With the necessary resources already in place by the end of a pre-implementation phase, the clinical implementation phase may aim to launch the co-designed implementation model in clinical practice starting from a hospital affiliated setting and gradually expanding towards primary care. Herein, we will present a pre-emptive panel-based model inspired by existing clinics in the USA such as the implementation project by the University of Colorado (Aquilante et al., 2020).

In the aforementioned project, UCHealth patients aged over 18 can sign an electronic consent form through their online UCHealth patient portal to allow both PGx research and the return of clinical results to the EMR. At the same time, an order to collect a sample at the patient’s next routine blood test is triggered. The sample drawn is then transferred to an affiliated Clinical Laboratory Improvement Amendments (CLIA) certified Biobank Laboratory for analysis by array tests, and various results may be returned to the patients and EMR, including PGx information, genetic diagnoses, carrier status, and predictors of disease risk (Aquilante et al., 2020).

Similarly, Quebec patients may be able to give their consent by signing an online consent form from their affiliated hospital which can be then integrated into the CDS system developed and prioritized in the pre-implementation phase. An ethics committee must review and approve the consent process before implementation. Patients are also suggested to discuss with their HCPs and ask all questions concerning the PGx research project.

Consenting patients will then be invited to provide, at their next routine blood test or anytime, a blood sample or a buccal swab at an affiliated center which will be transferred to a biobank laboratory such as Genome Quebec for genotyping or biobanking or both, depending on their consent form. The genotyping aspect is to improve clinical care while the biobanking aspect is to further research continuously in the field and the prevalence of population variants (Aquilante et al., 2020). PGx test results will then be integrated, through the platform developed in the pre-implementation phase, into the patient’s EMR. In the context of the province of Quebec, an example of such EMR would be the “*Dossier Santé Québec*” (DSQ), a pre-existing centralized communication platform storing a patient’s health information and facilitating its secure and timely sharing between authorized organizations and stakeholders. As a result, HPCs will have access to relevant patient PGx information whenever necessary (Dossier santé Québec DSQ, 2023).

With patient PGx data integrated into their EMRs, PCPs will be able to receive an electronic CDS alert with relevant PGx information such as the patient’s genotype, phenotype, and CPIC clinical guidelines in both French and English including necessary therapeutic recommendations when prescribing high-risk medications. Pharmacists, with their expertise in the field of medication, can support physicians with the prescription, review and provide explanations on the impact of PGx variants on the posology if requested including counselling patients with their PGx test result. In the event of incidental findings, patients can be referred to appropriate healthcare providers, e.g., genetic counsellors.

## Conclusion

With an increasing number of drug-gene interactions found, pharmacogenetics focuses on the utilization of a patient’s genetic variants to personalize treatment with increased therapeutic effect and decreased risk of ADRs. Twenty-seven medications are frequently cited in ADRs and approximately 60% of them are associated with at least one drug-metabolizing enzyme with known PGx variants (Mroz et al., 2021). As a randomized controlled trial shows a 30% decrease in clinically significant ADRs by implementing pre-emptive panel-based PGx-guided prescriptions (Oni-Orisan et al., 2023; Swen et al., 2023), we believe the rapid implementation of PGx is more than relevant.

While many barriers to PGx implementation have been raised, many solutions have also emerged over the years. Surveys also indicate the interest and willingness of patients and HCPs to engage in PGx which is in line with shared decision-making (The Kings Fund, 2011; Lemke et al., 2017). Therefore, while the establishment of a PGx testing program in the province of Quebec will be challenging, we believe it to be achievable. To address the cost of PGx testing, pre-emptive and panel-based PGx can be deployed to reduce the number of single-gene tests, thus improving cost-effectiveness (ODonnell et al., 2012; Bielinski et al., 2014; Van Driest et al., 2014; Klein et al., 2017; Blagec et al., 2022; Hayashi et al., 2022; Morris et al., 2022; Huang et al., 2023; McDermott et al., 2023; Swen et al., 2023) and with the added benefit of timelessness (ODonnell et al., 2012; Johnson et al., 2013; Bielinski et al., 2014; Hoffman et al., 2014; Rasmussen-Torvik et al., 2014; Van Driest et al., 2014; Rosenman et al., 2017; Van Der Wouden et al., 2017; Chumnumwat et al., 2019; Duarte et al., 2021; Albalwy et al., 2022; Hayashi et al., 2022; Morris et al., 2022; Huang et al., 2023; Kabbani et al., 2023; McDermott et al., 2023; Oni-Orisan et al., 2023). Technologies such as NGS available in Quebec at institutions such as Genome Quebec (Sequencing, 2023) can further decrease the cost of panel-based PGx testing to potentially a couple of USD (Huang et al., 2023). The reported lack of clinical guidelines can be remediated by organizations such as CPIC or DWPG (Relling and Klein, 2011; Swen et al., 2011) and by a proactive involvement of governmental instances such as INESSS to produce references to guidance tools. Furthermore, the development of CDS tools integrated into patients’ EMRs can aid clinicians in making efficient clinical decisions using patients’ PGx information (Relling and Klein, 2011; Swen et al., 2011). Laws such as the Genetic Non-Discrimination Acts of Canada are in place, preventing discrimination based on genetic information (De Denus et al., 2013; Branch, 2017; Hayashi et al., 2022; Oni-Orisan et al., 2023). Pre-existing platforms like PREVALIS can develop the digital ecosystem required for PGx clinical implementation while guaranteeing its ethical and medicolegal aspects (PREVALIS, 2023). Canadian initiatives like PANS and the previously existing PRIME PGx lay the foundation for online workshops and courses on PGx to remediate the need for educational resources (McDermott et al., 2023). Moreover, IPE workshops can be realistically organized as well by Quebec universities to answer the lack of PGx knowledge with the added benefit of improving interprofessional collaboration (Calinski et al., 2021; Lee et al., 2023).

In summary, the clinical implementation of PGx will optimize treatment and bring various benefits to patients and the Quebec healthcare system. PGx implementation should consider the incidence of ADR, cost-effectiveness, adherence of patients and HCPs to the guidelines, and tools’ efficiency as potential primary outcomes. We believe it will become a great assistance in achieving the universal objective of personalized and highly effective clinical care, minimizing toxicity. This implementation will serve to enhance standard practices rather than replace them. Upon a thorough examination of the available literature on the subject, we believe that Quebec possesses the essential resources to further advance its exploration into the implementation of pharmacogenetics testing in its hospitals and clinics. As interest and willingness to implement along with accessible and pre-existing resources to remediate barriers previously raised are both present, Quebec is well equipped for meaningful development in the field of PGx testing implementation. As such, we have amounted to a possible solution for its clinical implementation adapted to the resources and perspectives unique to the province. The next few years should be dedicated to applying, experimenting, and improving the implementation model. Initial efforts will involve smaller-scale applications, e.g., pilot and pre-implementation studies. Subsequently, these initiatives will be expanded to include primary care clinics before progressing to a provincial-level implementation.
